# Relationship between estimated and observed heparin sensitivity indices in cardiac and thoracic aortic surgery

**DOI:** 10.1186/s40981-023-00642-8

**Published:** 2023-08-05

**Authors:** Hitomi Nakatani, Mitsuru Ida, Taichi Kotani, Masahiko Kawaguchi

**Affiliations:** 1 Department Resource Nurse Center, Nara Medical University, 840 Shijo-Cho, Kashihara, Nara 634-8522 Japan; 2 Department of Anesthesiology, Nara Medical University, 840 Shijo-Cho, Kashihara, Nara 634-8522 Japan

**Keywords:** Heparin sensitivity index, Heparin resistance, Cardiopulmonary bypass, Activated clotting time

## Abstract

**Background:**

Little evidence exists on the relationship between the estimated heparin sensitivity index (HSI) based on commonly available clinical and laboratory data and observed HSI in the adult population. This retrospective study assessed the relationship between the observed and estimated HSIs.

**Methods:**

This study was conducted in an academic, single-institution setting. Patients aged ≥ 20 years who underwent cardiac and thoracic aortic surgery and requiring cardiopulmonary bypass were included. Clinical and laboratory data, including age, sex, and platelet count, were collected. The fibrinogen-albumin ratio index was calculated by dividing the fibrinogen value by the albumin value, multiplied by 10.The HSI was calculated using the formula: (activated clotting time after initial heparin administration—baseline activated clotting time)/initial heparin dose (IU/kg). The estimated HSI was based on the results of multiple regression analysis that included clinically relevant factors. The intraclass correlation coefficient between the observed and estimated HSIs was used to assess.

**Results:**

In total, 560 patients with valid activated clotting time (ACT) values after initial heparin administration were included in the final analysis to explore associated factors using the estimated HSI. Multiple regression analysis revealed that hemodialysis, platelet count, fibrinogen-to-albumin ratio index, baseline activated clotting time, and initial heparin dose were significantly associated with the HSI. The mean (standard deviation) observed and estimated HSIs were 1.38 (0.43) and 1.55 (0.13), respectively, with an intra-class correlation coefficient of 0.10.

**Conclusions:**

The correlation between the observed and estimated HSIs was low, and a formula with high accuracy for estimating the HSI is needed.

**Supplementary Information:**

The online version contains supplementary material available at 10.1186/s40981-023-00642-8.

## Introduction

Unfractionated heparin is the first choice in the initiation and maintenance of cardiopulmonary bypass (CPB) owing to its low cost, established monitoring method, and presence of a reliable antagonist, protamine. Several established methods for monitoring the effectiveness of heparin are available, but activated clotting time (ACT) is the most popular in the operating room [1, 2]. In cases of cardiac and thoracic aortic surgery patients requiring CPB, heparin resistance (HR) has been defined as failure to achieve the target ACT despite administering a standard dose of heparin. However, the target ACT and standard dose of heparin have differed among studies, resulting in incidences of HR ranging from 4% to 30% [2, 3].

ACT is theoretically affected by various factors, including medications, platelet count, fibrinogen level, and antithrombin level, and previous studies have stated that some of these factors are associated with HR [1–6]. Therefore, different levels of heparin sensitivity result in inter-individual variations in heparin doses for a target ACT for establishing CPB. The heparin monitoring device, Hepcon HMS Plus (Medtronic, Minneapolis, MN), enables the estimation of the heparin dose for achieving a target ACT with a single dose of heparin. However, two large-scale clinical studies in adult and paediatric populations found that the Hepcon HMS Plus failed to estimate the heparin dose for establishing CPB, and the heparin sensitivity measured using the device had poor agreement with the heparin sensitivity, based on clinical data [4–7]. Heparin sensitivity index (HSI) is the ACT time prolonged by administrating one unit of heparin converted per body weight and is calculated using the following formula: (ACT after initial heparin administration—baseline ACT)/ initial heparin dose (IU/kg). Little evidence is available on the relationship between the estimated heparin sensitivity index (HSI) based on commonly available clinical and laboratory data and the observed HSI in the adult population. Hence, this study aimed to assess the incidence of HR and its associated factors and to evaluate the relationship between the observed and estimated HSIs.

## Methods

### Ethics approval and consent to participate

This retrospective study was approved by the Institutional Review Board of Nara Medical University (Chairperson: Prof. MY; approval number: 3402; September 20, 2022). The need for informed consent was waived due to the retrospective nature of the study.

#### Inclusion and exclusion criteria

Patients aged ≥ 20 years who underwent cardiac and thoracic aortic surgery requiring CPB from January 2011 to July 2022 at a single academic center were eligible for this study. The exclusion criteria were as follows: 1) presence of coagulation disorders, 2) missing preoperative blood test data, 3) abnormally high activated partial thromboplastin time (APTT) (> 50 s) and prothrombin time international normalized ratio (PT-INR) (> 2.5), 4) requirement of heparin administration before full heparinization, 5) no ACT data at baseline or after full heparinization, and 6) missing data regarding heparin dose.

#### Data collection

Preoperative data including the patients’ age, sex, body mass index (BMI), current smoking habits, hypertension, symptomatic cerebral disease, hemodialysis, daily use of beta-blockers and digitalis, use of preoperative intravenous nitrate solution, specific diseases, including infective endocarditis and dissection of the aorta (acute and chronic), and laboratory data were collected (aspartate aminotransferase, alanine aminotransferase, platelet, fibrinogen, serum albumin, antithrombin, and serum creatinine levels, PT-INR, APTT). The fibrinogen-albumin ratio index (FARI) was calculated by dividing the fibrinogen value by the albumin value, multiplied by 10. Fibrinogen and albumin affect HR; however, both are components of serum proteins. FARI, used as an indicator for inflammation, treats these components as a single indicator to eliminate clinical multicollinearity [3, 5]. Intraoperative data, including surgical procedures (valve, coronary artery, aorta, and others), baseline ACT, initial heparin dose (U/kg), ACT after initial heparin administration, surgical duration, and blood loss volume, were recorded by the anaesthesiologist. The ACT in patients included in this study was measured using ACT plus (Medtronic, Minneapolis, MN).

#### Outcome

The primary outcome of this study was the correlation between the observed and estimated HSI. The secondary outcomes were the incidence of HR and its associated factors. In this study, HR was defined as an ACT of < 480 s under a heparin dose determined by the attending anaesthesiologist. The target ACT in our clinical setting was set in accordance with international guidelines [8]. Additionally, the reasons for failure to achieve an ACT ≥ 480 s were assessed.

#### Statistical analysis

Continuous variables were presented as means (standard deviation [SD]), and categorical data as numbers (%). The incidence of HR was calculated, and the 95% confidence interval (CI) was estimated using the Agresti–Coull method. Multiple logistic analysis was performed to estimate odds ratios for clinically relevant factors, including age, sex, hemodialysis, current smoking, platelet count, PT-INR, antithrombin, FARI, baseline ACT, and initial heparin dose (per unit/kg) [3–5]. Discrimination was assessed using the area under the receiver operating characteristic (ROC) curve. To estimate the HSI, multiple regression analysis was performed using clinically relevant factors described above, excluding those with an ACT > 999 s after initial intravenous heparin. Then, the correlation between observed and estimated HSI was assessed using the intra-class correlation coefficient. Bootstrapping (replications = 1000) was used to calculate the regression coefficient and 95% CI in the multiple logistic and regression analyses. All data were analyzed using SPSS v25.0 (IBM, Armonk, NY, USA), and *P* < 0.05 was considered statistically significant. As a post-hoc analysis required on the peer review process, a Bland–Altman plot was made for analyzing the agreement between two different HSI, which are observed and estimated HSI. Additionally, the comparison of each intra-class correlation coefficient between the observed and the estimated heparin sensitivity index by using FARI or fibrinogen only.

We assumed that the incidence of HR was 20% based on previous studies [2, 3]. When there were 10 covariates in the multiple logistic analysis, based on the minimal criterion of 10 events per predictor, at least 500 patients were required. Assuming that 15% of patients would have insufficient data, the required minimum number of cases was 589 in this study. This sample size included the one for multiple regression analysis.

## Results

**Fig. 1:**
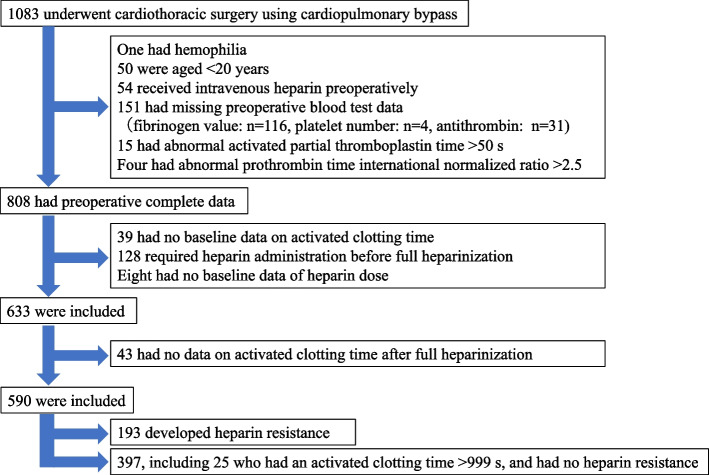
Patient selection flowchart

**Fig. 2:**
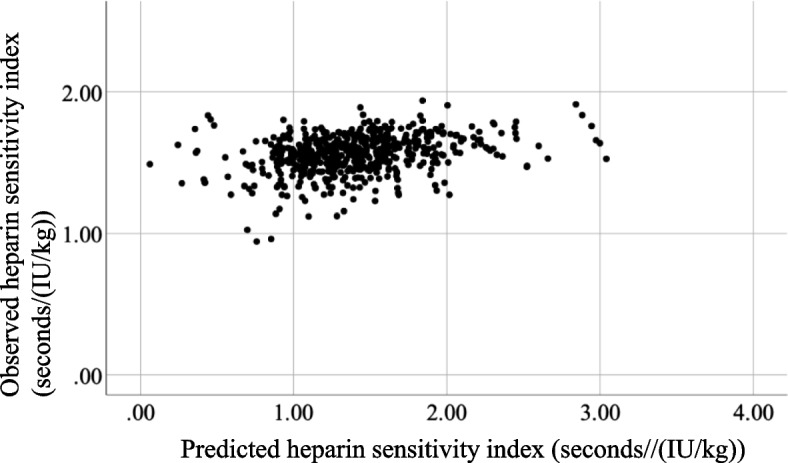
Relationship between predicted heparin sensitivity index and observed heparin sensitivity indices

Among the eligible patients, 590 had complete data (Fig. 1). The mean FARI score was 9.4 (4.8). The mean baseline ACT and initial heparin dose (IU/kg) were 154 (16) and 275 (29), respectively. HR occurred in 193/590 (32.7%, 95% CI: 29.0 − 36.6) patients. Table 1 shows the patient demographics and intraoperative data. Multiple logistic analysis revealed that hemodialysis, platelet count, FARI, baseline ACT, and initial heparin dose were statistically significant factors, with an area under the ROC curve of 0.70 (95% CI, 0.60–0.75) (Table 2). Multiple regression analysis, including 565 patients with valid ACT values after initial heparin administration and with an observed HSI 1.39 (0.4) seconds/(IU/kg) (Table 3), showed that the same factors as those in the multiple logistic analysis were significant for HSI (Table 4). The estimated HSI was calculated using the following formula: 1.803—0.215 × hemodialysis—0.011 × platelet—0.008 × fibrinogen albumin ratio index + 0.004 × baseline activated clotting time—0.002 × initial heparin dose. As a result, the mean estimated HSI was 1.55 (0.13) seconds/(IU/kg); A scatter plot of observed and predicted HSI shown in Fig. 2 and their intra-class correlation coefficient was 0.10 (95% CI 0.02 − 0.18, *p* = 0.007). The intra-class correlation coefficient between the observed and the estimated heparin sensitivity index by using and fibrinogen only was 0.02 (-0.06, 0.10) (*p* = 0.29) and a Bland–Altman plot is shown in Supplementary Figure 1.

**Table 1:** Patient demographics and intraoperative data (*n* = 590)

	Total (*n* = 590)	Heparin resistance (-) (*n* = 397)	Heparin resistance ( +) (*n* = 193)	*P* value
Age (yr)	68.6 (11.6)	68.6 (11.5)	68.6 (11.9)	0.98
Female	329 (55.8)	235 (59.2)	94 (48.7)	0.01
Body mass index (kg/m^2^)	23.3 (3.6)	23.6 (3.6)	22.8 (3.5)	0.007
Current smoking	37 (6.3)	25 (6.3)	12 (6.2)	> 0.99
Hypertension	418 (70.8)	287 (72.3)	131 (67.9)	0.28
Symptomatic cerebral disease	11 (1.9)	7 (1.8)	4 (2.1)	0.75
Hemodialysis	42 (7.1)	24 (6.0)	18 (9.3)	0.17
Daily medication				
Beta blocker	197 (33.4)	139 (35.0)	58 (30.1)	0.26
Digitalis	21 (3.6)	15 (3.8)	6 (3.1)	0.81
Intravenous nitrate solution	155 (26.3)	105 (26.4)	50 (25.9)	0.92
Specific disease				
Infective endocarditis	34 (5.8)	23 (5.8)	11 (5.7)	> 0.99
Dissection of aorta (acute and chronic)	47 (8.0)	31 (7.8)	16 (8.3)	0.87
Laboratory data				
Aspartate aminotransferase (IU/L)	31 (104)	34 (126)	25 (16)	0.3
Alanine aminotransferase (IU/L)	26 (111)	29 (134)	20 (21)	0.36
Platelet (104/μL)	19.6 (6.7)	18.9 (6.3)	21.0 (7.3)	< 0.001
Fibrinogen (mg/dL)	355 (133)	345 (124)	377 (147)	0.007
Serum albumin (d/dL)	3.9 (0.5)	3.9 (0.5)	3.9 (0.5)	0.47
Fibrinogen albumin ratio index	9.4 (4.8)	9.1 (4.3)	10.2 (5.7)	0.01
Antithrombin (%)	96 (17)	95 (16)	97 (17)	0.25
Serum creatinine (mg/dL)	1.49 (1.9)	1.39 (1.6)	1.71 (2.4)	0.05
Prothrombin time-international normalized ratio	1.11 (0.2)	1.12 (0.2)	1.09 (0.2)	0.21
Activated partial thromboplastin time (s)	29.6 (4.6)	30.1 (4.7)	28.5 (4.1)	< 0.001
Intraoperative data				
Surgical procedure*				0.005
Valve	277 (46.9)	191 (48.1)	86 (44.6)	
Coronary artery	127 (21.5)	99 (24.9)	28 (14.5)	
Aorta	198 (33.6)	122 (30.7)	76 (39.4)	
Others	79 (13.4)	46 (11.6)	33 (17.1)	
Baseline activated clotting time (s)	154 (16)	156 (16)	149 (17)	< 0.001
Initial heparin dose (u/kg)	275 (29)	279 (29)	269 (28)	< 0.001
Activated clotting time after initial heparin administration (s)	555 (150)	624 (134)	415 (57)	< 0.001
Surgical duration (min)	415(148)	418 (147)	409 (150)	0.48
Blood loss volume (mL)	1732 (1892)	1768 (1871)	1659 (1937)	0.51

Mean (standard deviation) or number (%)

*Number of patients who underwent multiple surgical procedures (e.g., aortic valve replacement and coronary artery bypass grafting)

**Table 2:** Results of multiple logistic analysis calculating odds ratios for heparin resistance (*n* = 590)

	Odds ratio (95% Confidence interval)	*P* value
Age	1.00 (0.98–1.02)	0.48
Female	0.71 (0.48–1.05)	0.09
Hemodialysis	2.35 (1.15–4.79)	0.01
Current smoking	0.82 (0.37–1.80)	0.62
Platelet	1.04 (1.01–1.08)	0.003
Prothrombin time-international normalized ratio	0.66 (0.27–1.59)	0.36
Antithrombin	1.00 (0.99–1.01)	0.54
Fibrinogen albumin ratio index	1.05 (1.01–1.10)	0.01
Baseline activated clotting time	0.97 (0.95–0;98)	< 0.001
Initial heparin dose (per unit/kg)	0.98 (0.97–0.99)	< 0.001

The explanatory model based on these variables had an area under the receiver operating characteristic curve of 0.70 (95% confidence interval, 0.60 − 0.75)

**Table 3:** Demographics and intraoperative data for patients with measurable activated clotting time following initial heparin administration (*n* = 565)

	Total (*n* = 565)	Heparin resistance (-) (n-372)	Heparin resistance ( +) (n-193)	*P* value
Age (yr)	68.7 (11.4)	68.8 (11.2)	68.6 (11.9)	0.84
Female	314 (55.6)	220 (59.1)	94 (48.7)	0.02
Body mass index (kg/m^2^)	23.3 (3.6)	23.6 (3.6)	22.8 (3.5)	0.01
Current smoking	37 (6.5)	25 (6.7)	12 (6.2)	0.86
Hypertension	403 (71.3)	272 (73.1)	131 (67.9)	0.2
Symptomatic cerebral disease	10 (1.8)	6 (1.6)	4 (2.1)	0.74
Hemodialysis	41 (7.3)	23 (6.2)	18 (9.3)	0.17
Daily medication				
Beta blocker	184 (32.6)	126 (33.9)	58 (30.1)	0.39
Digitalis	21 (3.7)	15 (4.0)	6 (3.1)	0.64
Intravenous nitrate solution	149 (26.4)	99 (26.6)	50 (25.9)	0.92
Specific disease				
Infective endocarditis	33 (5.8)	22 (5.9)	11 (5.7)	> 0.99
Dissection of aorta (acute and chronic)	46 (8.1)	30 (8.1)	16 (8.3)	> 0.99
Laboratory data				
Aspartate aminotransferase (IU/L)	30 (105)	33 (129)	25 (16)	0.39
Alanine aminotransferase (IU/L)	26 (113)	29 (138)	20 (21)	0.38
Platelet (104/μL)	19.7 (6.7)	19.0 (6.3)	21.0 (7.3)	0.001
Fibrinogen (mg/dL)	359 (133)	349 (123)	377 (147)	0.02
Serum albumin (d/dL)	3.9 (0.5)	3.9 (0.5)	3.9 (0.5)	0.36
Fibrinogen albumin ratio index	9.5 (4.8)	9.2 (4.3)	10.2 (5.7)	0.02
Antithrombin (%)	96.5 (16.9)	96.2 (16.6)	97.2 (17.4)	0.5
Serum creatinine (mg/dL)	1.50 (1.9)	1.39 (1.6)	1.71 (2.4)	0.06
Prothrombin time-international normalized ratio	1.11 (0.2)	1.12 (0.2)	1.09 (0.2)	0.22
Activated partial thromboplastin time (s)	29.4 (4.4)	29.9 (4.5)	28.5 (4.1)	< 0.001
Intraoperative data				
Surgical procedure*				0.003
Valve	266 (47.1)	180 (48.4)	86 (44.6)	
Coronary artery	121 (21.4)	93 (25.0)	28 (14.5)	
Aorta	184 (32.6)	108 (29.0)	76 (39.4)	
Others	77 (13.6)	44 (11.8)	33 (17.1)	
Baseline activated clotting time (s)	153 (16)	155 (15)	149 (17)	< 0.001
Initial heparin dose (u/kg)	276 (28)	280 (26)	269 (28)	< 0.001
Activated clotting time after initial heparin administration (s)	536 (121)	599 (95)	415 (57)	< 0.001
Heparin sensitivity index (seconds/(IU/kg))	1.39 (0.4)	1.59 (0.3)	1.00 (0.2)	< 0.001
Surgical duration (min)	410 (142)	411 (138)	409 (150)	0.91
Blood loss volume (mL)	1703 (1893)	1726 (1872)	1659 (1937)	0.69

Mean (standard deviation) or number (%)

*Number of patients who underwent multiple surgical procedures (e.g., aortic valve replacement and coronary artery bypass grafting)

**Table 4:** Results of multiple regression analysis for the heparin sensitivity index (*n* = 565)

	Regression coefficient (β) (95% confidence interval (lower limit, upper limit))	*P* value
Constant	1.803 (1.222, 2.384)	< 0.001
Age	< 0.001 (-0.003, 0.003)	0.88
Female	0.068 (-0.003, 0.140)	0.06
Hemodialysis	-0.215 (-0.351, -0.079)	0.002
Current smoking	-0.033 (-0.172, 0.106)	0.64
Platelet	-0.011 (-0.0016, -0.005)	< 0.001
Prothrombin time-international normalized ratio	0.016 (-0.131, 0.163)	0.82
Antithrombin	-0.001 (-0.004, 0.001)	0.18
Fibrinogen albumin ratio index	-0.008 (-0.016, < 0.000)	0.04
Baseline activated clotting time	0.004 (0.001, 0.006)	0.001
Initial heparin dose (per unit/kg)	-0.002 (-0.003, -0.001)	0.001

Of the 193 patients who failed to achieve an ACT of ≥ 480 s after initial heparin administration, 86 received a second dose of heparin. Of these, only one patient was administered recombinant human antithrombin after the second heparin administration.

## Discussion

Several previous studies have shown factors associated with HR, although they had no data the dose of heparin for the establishment of CPB. Other studies have attempted unsuccessfully to use specific monitoring device to predict the dose of heparin required to establish a CPB; therefore, this retrospective study aimed to assess the incidence of HR and its associated factors and evaluate the relationship between the observed and estimated HSIs using clinically available data. We included patients who underwent elective and emergency cardiovascular surgeries requiring CPB. The incidence of HR was 32.7%. Patients who depended on hemodialysis had a high platelet count, high FARI, and low baseline ACT, and those who received a small initial heparin dose had a higher likelihood of developing HR. Although the same factors were also associated with HSI, the correlation between the observed and predicted HSI was low.

The incidence of HR in this study, defined as an ACT of < 480 s after initial heparin administration, was higher than that in previous studies [2, 3]; this difference can be attributed to the different definitions used in each study.

Although specific diseases such as infective endocarditis and chronic thoracic aortic dissection were significantly associated with HR in previous studies, we hypothesized that patients with these diseases were more likely to have inflammation; thus, we planned to include only the inflammation index, namely FARI, in advance [3, 5]. As a result, FARI and some associated factors shown in previous studies were also included in this study [1–6], indicating that our cohort was not unique but general.

The factors associated with HR have been evaluated as described above; however, our results do not provide a heparin dose to achieve a target ACT. In our cohort, 104 patients did not receive additional heparin, possibly because of the 5000 IU of unfractionated heparin in the CPB prime solution. In most of the remaining patients, an additional heparin administration provided an ACT of ≥ 480 s. This suggests that a large amount of heparin leads to an ACT ≥ 480 s. However, large doses of heparin require large amounts of protamine. Considering the high incidence of protamine allergies and the fact that protamine can cause dose-dependent allergic-like reactions, minimal amounts of heparin should be administered [9, 10]. Unfortunately, although the same factors associated with HR were statistically significant for HSI, a low intraclass correlation coefficient between the observed and predicted HSIs was observed. This might be due to the limitation of using ACT for monitoring the underlying multifactorial causes of HR [1, 2, 11]. Surprisingly, even when using a device that estimates the heparin dose, 17% of the patients failed to achieve a target ACT (350 s), indicating the difficulty in estimating the HSI [7].

This study had some limitations. First, clinically available data were limited owing to the retrospective nature of the study. Including more clinical data would contribute to a more accurate prediction. In the future, we hope to identify stronger predictors. Second, this was a single-center study; therefore, the results lack generalizability.

## Conclusions

In conclusion, hemodialysis, platelet count, FARI, baseline ACT, and initial heparin dose were associated with HR and the HSI in adult patients who underwent elective and emergency cardiac and thoracic aortic surgery involving CPB; however, the relationship between the estimated and observed HSIs was low. Future studies are needed to determine the amount of heparin needed to prevent delay during CPB.

### Supplementary Information

**Additional file 1: Supplementary Figure 1.** Bland-Altman plot of predicted heparin sensitivity index and observed heparin sensitivity indices. Bland-Altman plot of heparin sensitivity index. Dashed line denotes mean of difference and 95％ limits of agreement (±1.96＊standard deviation of difference).

## Data Availability

We can provide our data according to readers’ request.

## Abbreviations

ACT: Activated clotting time
APTT: Activated partial thromboplastin time
BMI: Body mass index
CI: Confidence interval
CPB: Cardiopulmonary bypass
FARI: Fibrinogen-albumin ratio index
HR: Heparin resistance
HSI: Heparin sensitivity index
PT-INR: Prothrombin time international normalised ratio
ROC: Receiver operating characteristic
SD: Standard deviation
