# Supplementary figures and images for: Relationship between estimated and observed heparin sensitivity indices in cardiac and thoracic aortic surgery
Source: JA Clin Rep. 2023 Aug 5;9:50. doi: 10.1186/s40981-023-00642-8 (PMC10403474; doi:10.1186/s40981-023-00642-8)

## Slide 1
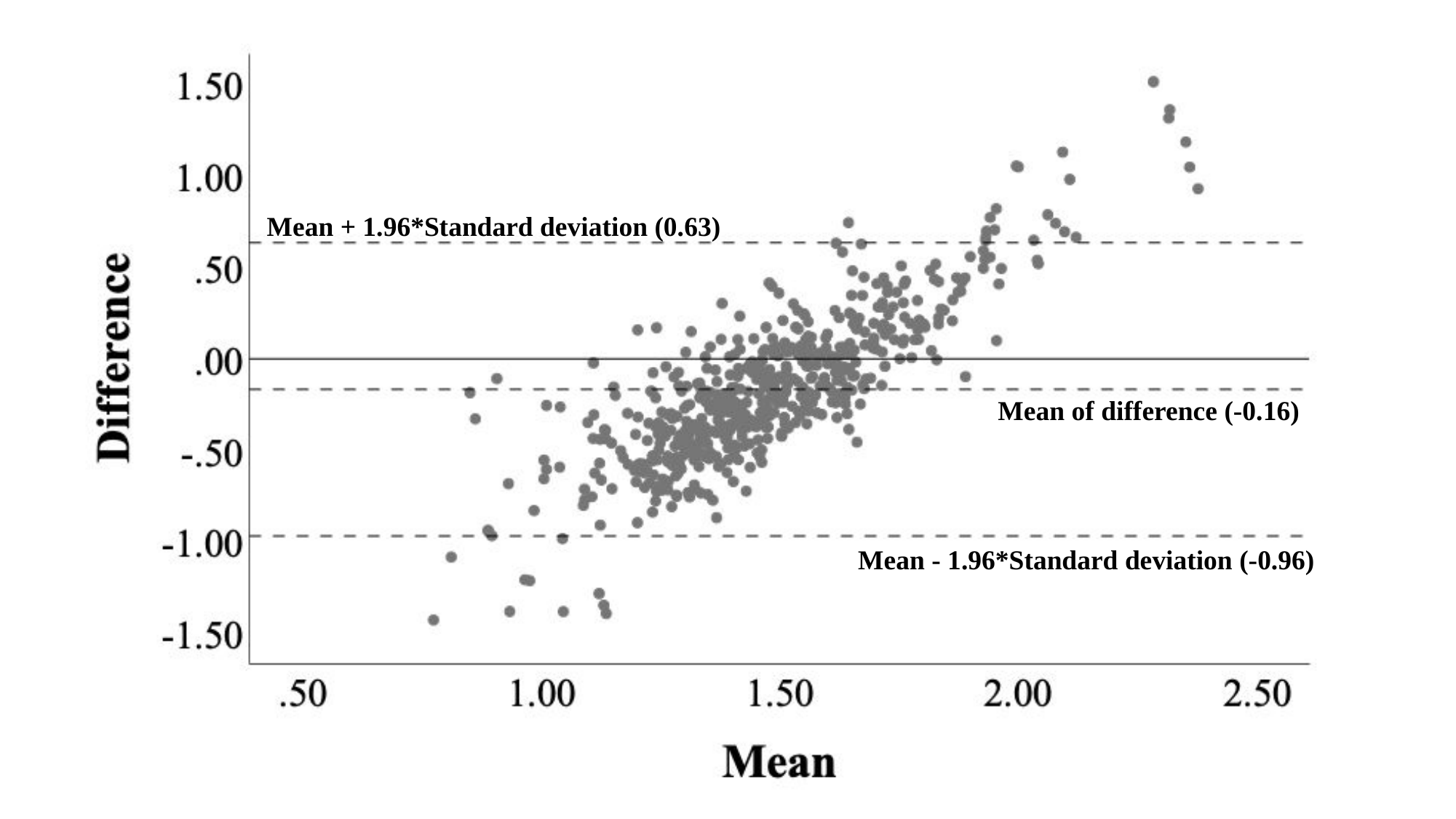

Mean + 1.96*Standard deviation (0.63)
Mean of difference (-0.16)
Mean - 1.96*Standard deviation (-0.96)

Supplement: Supplementary file 1 — Additional file 1: Supplementary Figure 1. Bland-Altman plot of predicted heparin sensitivity index and observed heparin sensitivity indices. Bland-Altman plot of heparin sensitivity index. Dashed line denotes mean of difference and 95％ limits of agreement (±1.96＊standard deviation of difference). [file 40981_2023_642_MOESM1_ESM.pptx]
